# The composite water and solute transport of barley (*Hordeum vulgare*) roots: effect of suberized barriers

**DOI:** 10.1093/aob/mcw252

**Published:** 2017-01-08

**Authors:** Kosala Ranathunge, Yangmin X. Kim, Friedrich Wassmann, Tino Kreszies, Viktoria Zeisler, Lukas Schreiber

**Affiliations:** 1 Department of Ecophysiology, Institute of Cellular and Molecular Botany, University of Bonn, Kirschallee 1, D-53115 Bonn, Germany; 2 Department of Soil Hydrology, George-August-University of Göttingen, Büsgenweg 2, D-37077 Göttingen, Germany

**Keywords:** Apoplast, barley, composite transport, hydraulic conductivity, osmometer model, reflection coefficient, solute permeability

## Abstract

**Background and Aims** Roots have complex anatomical structures, and certain localized cell layers develop suberized apoplastic barriers. The size and tightness of these barriers depend on the growth conditions and on the age of the root. Such complex anatomical structures result in a composite water and solute transport in roots.

**Methods** Development of apoplastic barriers along barley seminal roots was detected using various staining methods, and the suberin amounts in the apical and basal zones were analysed using gas chromatography–mass spectometry (GC-MS). The hydraulic conductivity of roots (*Lp*_r_) and of cortical cells (*Lp*_c_) was measured using root and cell pressure probes.

**Key Results** When grown in hydroponics, barley roots did not form an exodermis, even at their basal zones. However, they developed an endodermis. Endodermal Casparian bands first appeared as ‘dots’ as early as at 20 mm from the apex, whereas a patchy suberin lamellae appeared at 60 mm. The endodermal suberin accounted for the total suberin of the roots. The absolute amount in the basal zone was significantly higher than in the apical zone, which was inversely proportional to the *Lp*_r_. Comparison of *Lp*_r_ and *Lp*_c_ suggested that cell to cell pathways dominate for water transport in roots. However, the calculation of *Lp*_r_ from *Lp*_c_ showed that at least 26 % of water transport occurs through the apoplast. Roots had different solute permeabilities (*P*_sr_) and reflection coefficients (*σ*_sr_) for the solutes used. The *σ*_sr_ was below unity for the solutes, which have virtually zero permeability for semi-permeable membranes.

**Conclusions** Suberized endodermis significantly reduces *Lp*_r_ of seminal roots. The water and solute transport across barley roots is composite in nature and they do not behave like ideal osmometers. The composite transport model should be extended by adding components arranged in series (cortex, endodermis) in addition to the currently included components arranged in parallel (apoplastic, cell to cell pathways).

## INTRODUCTION

In recent years, there has been an increasing amount of interest in modelling root hydraulics. This interest is due to the fact that within the soil–plant–air continuum (SPAC), the water taken up by plant roots either can be used for plant growth and development or can be lost by transpiration (Kramer, 1983). The discovery of aquaporins (AQPs) in the early 1990s suggested that they were a major regulatory component for water transport across cell membranes within the SPAC (Maurel, 1997; Tyerman *et al.*, 1999; Maurel and Chrispeels, 2001). However, this picture has changed in light of further quantitative data from pressure probes concerning the hydraulic properties of individual root cells and the overall hydraulic conductivities of entire roots (Zhu and Steudle, 1991; Steudle and Peterson, 1998). In many of these studies, the results have indicated that the apoplastic path contributes to water transport, even across the endodermis. There are exceptions to these results, however, as reported in young roots of barley (Steudle and Jeschke, 1983), bean (Steudle and Brinckmann, 1989) and *Arabidopsis thaliana* (Ranathunge and Schreiber, 2011). In these plants, cell to cell water transport dominated in roots, suggesting that AQPs were the major influence on water transport. The existence of two pathways, along with composite transport, would provide some explanation for the observed variance in root hydraulic conductivity (*Lp*_r_) besides the action of AQPs (Brouwer, 1954; Weatherley, 1982; Kramer and Boyer, 1995). Regulation of *Lp*_r_ has been discussed in terms of the variable contributions of different pathways to the overall water flow in response to stresses such as drought, which affect root anatomy as well as water channel activity (Vandeleur *et al.*, 2009). This discussion is focused on the interaction between the two parallel pathways: the cell to cell and the apoplastic.

The simple composite transport model of the root, outlined in the previous paragraph, has been recently shown to be incomplete, because it does not include the root’s water storage capacity. This factor may be important when considering transient effects and thick roots (Meyer *et al.*, 2011). More importantly, the model usually considers only parallel pathways. In reality, roots contain additional transport components, such as the cortex and the stele, which are arranged in series. The endodermis, in particular, is known to be a substantial barrier for both water and solutes. On the other hand, the axial hydraulic resistance is usually a component of minor importance (Frensch and Steudle, 1989).

Recently, Knipfer and Fricke (2010) used root pressure probes to repeat some of the osmotic experiments conducted by Steudle and Jeschke (1983), using NaCl as the osmotic solute. The authors concluded that the reflection coefficients of the roots (*σ*_sr_) were very close to unity, suggesting that the roots behaved like ideal osmometers. These results were most probably due to an endodermis that was impermeable to the solute. This observation differed from the earlier results of Steudle and Jeschke (1983) for barley and for a number of other plant species (Steudle and Peterson, 1998, and references therein). Knipfer and Fricke (2010) concluded from their results that the cell to cell component of water transport, rather than the apoplastic component, was dominant in barley, confirming the previous data of Steudle and Jeschke (1983) that compared the cellular and overall root *Lp*_r_ by pressure probe measurements.

In the present study, we critically investigate the proposed cell to cell transport model of barley roots (Steudle and Jeschke, 1983) by combining anatomical, biochemical and physiological studies at the cellular and root level. We also determine how suberized barriers in the cell walls affect water transport, in addition to the extended measurements of the permeability patterns of these roots using several electrolytes and non-electrolytes as test solutes. We also propose certain modifications/additions to the accepted composite transport model, in which certain parameters should be added. These modifications would explain the rather low values of *σ*_sr_ in the presence of the low permeability coefficients (*P*_sr_) found in the roots of some other plant species during the osmotic experiments.

## MATERIALS AND METHODS

### Plant material and growth conditions

Caryopses of barley (*Hordeum vulgare* L. cv. ‘Golf’, Scottish Crop Research Institute) were germinated in the dark at 23 °C on filter paper moistened with a 0·5 mm CaSO_4_ solution. Six days later, the seedlings were transferred into a hydroponic system containing modified half-strength Hoagland solution in a climatic chamber (Fricke and Peters, 2002). The plants used in the experiments were grown for 16–20 d, including the germination period. At this stage, the plants had 3–4 developed leaves and 6–7 seminal roots. The maximum length and average diameter of the seminal roots varied between 70 and 140 mm and 0·4 and 0·6 mm, respectively.

### Histochemical detection of Casparian bands and suberin lamellae in roots

The seminal roots were cross-sectioned at distances of 10, 20, 30, 40, 50, 60 and 100 mm from the root apex. To detect the development of Casparian bands (CBs), the sections were stained with 0·1 % (w/v) berberine hemisulphate and 0·5 % (w/v) aniline blue (Brundrett *et al.*, 1988). The suberin lamellae were stained with lipophilic 0·01 % (w/v) Fluorol Yellow 088 (Brundrett *et al.*, 1991). The aliphatic suberin in cell walls was detected by yellow fluorescence under ultraviolet light (filter set: exciter filter, G 365; chromatic beam splitter, FT 395; barrier filter, LP 420). To detect the number of cell layers in the cortex and the cell dimensions, several cross-sections were stained with 0·05 % (w/v) Toludine blue O.

### Chemical analysis of the root suberin

The seminal roots were divided into two zones. Zone-I was the younger part of the root, without laterals, which included the growing root tip. This zone was identical to the end segments/apical part of roots used in the root pressure probe measurements. The average length of Zone-I was 60 ± 15 mm (*n* = 15). Zone-II was the mature half of the root, towards the base, and included lateral roots. The length of the mature zone was 50 ± 12 mm (*n* = 15). Root segments were enzymatically digested to remove cellulose and pectins from the cell walls (Zeier and Schreiber, 1997), and the steles were isolated along with the suberized endodermis. The isolated cell wall samples were then purified, dried and subjected to transesterification to release suberin monomers according to the procedures of Kolattukudy and Agrawal (1974). Gas chromatographic analysis and mass spectrometric identification of the derivatized degradation products were performed as described by Zeier and Schreiber (1997, 1998). The amounts were calculated for the unit surface area of the roots. Four replicates were used for each root zone.

### Measurement of hydraulic conductivity of roots (*Lp*_r_) and root cortical cells (*Lp*_c_) using pressure probes

The *Lp*_r_ of the end segments/apical part of the seminal roots (Zone-I; length: 50–75 mm) and total seminal roots (length: approx. 200 mm) was measured separately using a root pressure probe as earlier described by Steudle *et al.* (1987). Stable root pressure (*P*_r_) developed within 2–4 h after fixing the roots to the pressure probe. In the hydrostatic experiments, water flows were induced by moving the meniscus either forward to induce exosmotic water flow or backward to induce endosmotic water flow. The resulting hydrostatic relaxation curves were composed of two exponential phases brought about by different rates of changes of *P*_r_ over time. The initial rapid phase covered about 85 % of the entire pressure (volume) curve; followed by a slow reversible phase related to the concentration polarization effects at the endodermis (Steudle and Frensch, 1989; Hose *et al.*, 2000). The initial phase (see Fig. 3A) was used to measure the half-time of water exchange, *t*_1/2_^w^; and the hydrostatic hydraulic conductivity (*Lp*_r_) was determined from the rate constant of water exchange (*k*_wr_) (Steudle *et al.*, 1987, Steudle, 1989):

(1) $$k_{wr}=\frac{\ln(2)}{t_{1/2}^{w}}=Lp_{r}\times A_{r}\times \beta .$$

Here *β* (=*ΔP*_r_/*ΔV*_s_) is the elastic coefficient of the measuring system, ln(2) is the natural logarithm of 2 or 0·693, and *A*_r_ is the surface area of the conductive part of the root, which was approx. 85 % of the total surface area. This part had dead and functional (conductive) xylem vessels with lignified cell walls. The non-conductive root tip part (hydraulically isolated zone), which was approx. 15 mm long, was not taken into account for surface area calculations. This part did not have mature and functional xylem vessels, which were filled with the cytoplasm. Ten replicates were used for each measurement.

In the osmotic experiments, the nutrient solution in the external medium was rapidly exchanged by 30 mm (approx. 59 mOsmol kg^–1^) NaCl, a permeating solute (Fig. 3B). The osmotic *Lp*_r_ was calculated from the rate constant of the water phase of the biphasic osmotic root pressure relaxations (half-time of the first phase; Fig. 3B). Both experiments were conducted in two ways. In the first, the root medium was rapidly stirred to minimize the external unstirred layers, while in the second, the medium was kept stagnant without stirring to determine the effect of external unstirred layers on water permeability. Stirring was achieved by rapidly bubbling the medium with air (Ye and Steudle, 2006). During stagnant (unstirred) conditions, the bubbling was stopped.

The hydraulic conductivity of root cortical cells (*Lp*_c_) was measured by a cell pressure probe, as has been done for several different types of higher plant tissues, including roots (e.g. Azaizeh *et al.*, 1992; Steudle, 1993; Wan *et al.*, 2004; Kim and Steudle, 2007). Excised roots were mounted on a metal sledge carefully holding them by magnetic bars. The nutrient solution, used to grow plants in the hydroponic culture, ran along the roots using a recirculation system. Mid cortical cells of roots (third or fourth cell layer from the outside) were punctured at approx, 50 mm from the apex using an oil-filled microcapillary (average tip diameter: approx, 6 μm) to measure the cell *Lp*_c_. Once the cell was punctured, a meniscus formed between the cell sap and oil. Hydrostatic pressure relaxations were performed by gently moving the meniscus forward and backward to inject water into and out of the cells, respectively. Successful cell pressure probe experiments resulted in measuring cell turgor (*P*), the volumetric elastic modulus (*ε*) and the half-time of water exchange of individual cortical cells (*tc*_1/2_^w^). The *ε* was determined by the change in pressure (d*P*) according to the relative volume change (d*V/V*) by:

(2) $$\varepsilon =V\frac{dP}{dV}\;\approx \;V\frac{\Delta P}{\Delta V}$$

The hydraulic conductivity of the cell membrane (*Lp*_c_) was calculated using measured *tc*_1/2_^w^, *ε* and calculated mean values of cell volume (*V*) and cell surface area (*A*) from the following equation:

(3) $$Lp=\frac{V}{A}\;\frac{\ln(2)}{tC_{1/2}^{w}}\;\frac{1}{\varepsilon +\pi^{i}}.$$

The osmotic pressure of the cells (*π^i^*) was approximated by the stationary turgor pressure (*P*) of the cortical cells in the nutrient solution. Cell volume (*V*) and cell surface area (*A*) was calculated from the length and the diameter of cortical cells, assuming they are cylindrical in shape.

### Measurement of solute permeabilities (*P*_sr_) and reflection coefficients (*σ*_sr_) of roots

Several solutes which tend to have very different membrane permeabilities were used to measure the permeabilities (*P*_sr_) and reflection coefficients (*σ*_sr_) of the roots. Ethanol (100 mm = 100 mOsmol kg^–1^) was used as a solute that rapidly permeated the plasma membrane (Steudle and Tyerman, 1983; Tyerman and Steudle, 1984) and the root cylinder. NaCl (30 mm = 59 mOsmol kg^–1^), KCl (30 mm = 56 mOsmol kg^–1^) and mannitol (40 mm = 40 mOsmol kg^–1^) were used as less permeating solutes, while sucrose (60 mm = 60 mOsmol kg^–1^) and the 1:4 monovalent cation salt, K_4_[Fe(CN)_6_] (8 mm = 26 mOsmol kg^–1^) were used as virtually non-permeating solutes through the membranes. The osmotic concentration of solutes in the nutrient solution had an osmolality of 16 mOsmol kg^–1^. The *P*_sr_ of each solute was determined separately by the rate constant of solute exchange [*k*_sr = _ ln(2)/*t*^s^_1/2_] using the second phases in biphasic relaxations of osmotic experiments as given by Steudle *et al.* (1987):

(4) $$k_{sr}=\frac{\ln(2)}{t_{1/2}^{s}}=\frac{A_{r}\times P_{sr}\;}{V_{x}}.$$

In this equation, *t*_1/2_^s^ is the half-time of solute exchange and *V*_x_ is the volume of functional xylem in the root system, which is approx. 1 % of the total root volume, as measured from the cross-sections. The total root volume was calculated using the conductive length and the diameter of the root. The osmotic concentrations of the tested solutes were measured using a freezing point depression osmometer (Osmomat 030; Gonotec, Berlin, Germany).

The *σ*_sr_ values of the test solutes used in the *P*_sr_ measurements were calculated from the following equation:

(5) $$\sigma_{sr}=\frac{\Delta P_{r}}{\Delta \pi_{s}^{o}}\;\;\text{exp}\;(k_{sr}\times t_{\min}),$$

where Δ*P*_r_ is the maximum change in root pressure caused by changes in the osmotic pressure of the medium (Δ*π*_s_° = RT × C_s_; R = universal gas constant, T = absolute temperature, C_s_ = concentration of solute ‘s’ in the medium) and *t*_min_ is the time required to reach the minimum root pressure. The exponential term on the right side of eqn (2) is larger than unity and corrects for the solute flow during the time interval of *t*_min_. However, for non-permeating solutes, the curve was monophasic and the second phase or solute phase was missing. Hence, it was not possible to determine the exponential component of *k*_sr_ = ln(2)/*t*^s^_1/2_. For such solutes, *σ*_sr_ was calculated from the Δ*P*_r_, the maximum change in root pressure, caused by changes in the osmotic pressure of the medium (Kedem and Katchalsky, 1963*a*, *b*). For a semi-permeable membrane, which holds that *σ*_sr_ = 1, the addition of 40 mOsmol kg^–1^ would result in a pressure drop of 0·1 MP (1 bar). Since the osmotic pressure of the solutes in the medium was known, it was possible to calculate *σ*_sr_ for non-permeating solutes.

After each measurement, the proper function of the seal was checked by cutting off the root at the seal. When the xylem of the root remained open, there was a drastic decrease in the half-times (by at least one order of magnitude) and an increase in hydraulic conductance after the cut. If these results were not observed, the experiment was discarded.

### Measurement of root axial resistance by cutting experiments

To determine the maturity and functionality of xylem vessels and the development of axial resistance along the root axis, excised roots were attached to the root pressure probe (as described above). Once roots attained steady-state root pressures, successive cuts were made from the apex (at 5, 15 and 50 mm) using a sharp double-edged razor blade (Fig. 5). Once mature xylem vessels were cut, the root pressure (*P*_r_) quickly dropped to zero. Hydrostatic pressure relaxations were also performed between cuts to determine changes in the axial hydraulic resistance. Since the hydraulic capacitance (Δ*V*_s_/Δ*P*_r_) of the system remained constant, the recorded changes in *t*_1/2_^w^ directly reflected changes in the axial hydraulic resistance of roots (*R*; MPa s m^–4^).

(6) $$R=\frac{1}{Lp_{r}\times l_{rl}},$$

where *Lp*_r_ is the root hydraulic conductance (m^3^ MPa^–1^ s^–1^) and *l*_rl_ (m) is the remaining length of the root after each cut.

### Statistical analysis

Data were normally distributed and have been presented in figures and tables as means ± s.d. The Student’s *t*-test was employed to compare the means of the water permeabilities, while an analysis of variance (ANOVA) with the least significant difference (LSD) test was used to compare the means of *P*_sr_ and *σ*_sr_ for the different solutes used in the experiments. All statistical analyses were conducted with a confidence level of 95 %.

## RESULTS

### Root anatomy: development of Casparian bands and of suberin lamellae

**Fig. 1.**
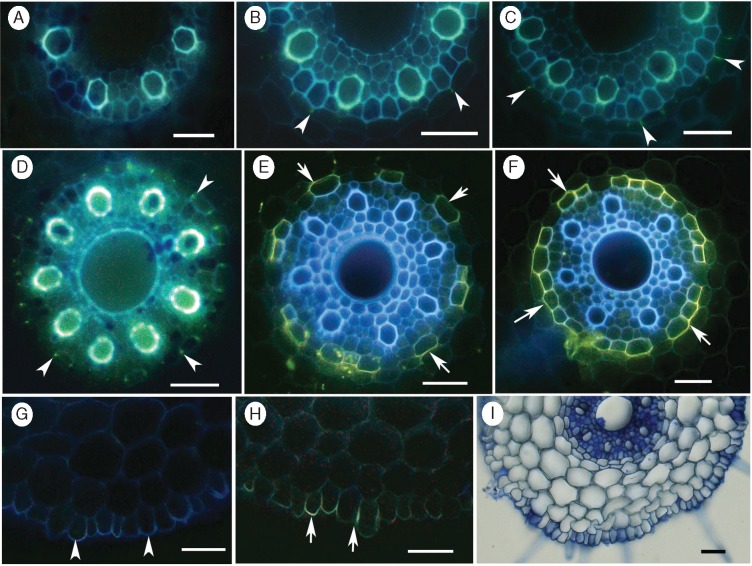
Appearance of Casparian bands (CBs) and suberin lamellae (SL) in the endodermis, and presence of suberin in the rhizodermis of barley roots. Freehand cross-sections of seminal roots of 16- to 20-day-old barley plants stained with either berberine–aniline blue (A-D) or lipophilic fluorochrome, Fluorol yellow 088 (E, F and H). At 10 mm from the apex, no CBs appeared in the endodermis (A), whereas, ‘dot-like’ bands, indicated by a faint green fluorescence, appeared at 20 mm from the apex (B; arrowheads). Continuous bands with intense yellowish green fluorescence in the radial walls at 30 (C) and 40 mm (D) from the tip, respectively. Stained SL appeared as a yellow, patchy ring in the endodermis (arrows) at 60 mm (E), but as a complete lamellae ring with bright yellow fluorescence (arrows) at 100 mm from the apex (F). Autofluorescence (G) and greenish yellow fluorescence (H) in the radial and outer tangential walls of the rhizodermis at 100 mm from the apex. Section stained with Toluidine blue O, showing only four cortical cell layers in the cortex (I). Scale bars = 50 µm.

Casparian bands in the endodermis were detected by a yellowish green fluorescence in the radial cell walls after staining with berberine–aniline blue (Fig. 1A–D). No CBs were detected at 10 mm from the apex (Fig. 1A). The bands first appeared as ‘dots‘, indicated by a faint green fluorescence, at 20 mm from the apex (Fig. 1B; arrowheads). At 30 and 40 mm, continuous bands with intense yellowish green fluorescence were observed in the radial walls of the endodermis (Fig. 1C, D; arrowheads).

The suberin lamellae (SL) were detected by an intense, bright yellow fluorescence in the cell walls after staining cross-sections with fluorol yellow 088 (Fig. 1E, F). At 60 mm from the apex, the stained SL was visible as a yellow, patchy ring in the endodermis (Fig. 1E; arrows). About 50 % of the endodermal cells were passage cells without lamellae. At 100 mm, well-developed SL with bright yellow fluorescence were apparent, and the passage cells accounted for <10 % (Fig. 1F). No exodermis was detected in the hydroponically grown barley roots. Even at 100 mm from the apex, neither CBs (Fig. 1G) nor SL (Fig. 1H) developed in the hypodermis (see Supplementary data, Fig. S1). However, clear autofluorescence (Fig. 1G) and yellow green fluorescence (Fig. 1H) in the outer tangential walls of the rhizodermis suggested the presence of ‘diffuse suberin’ (see Peterson *et al.*, 1978). The cross-section, stained with Toludine blue O, confirmed that there were four cortical cell layers present in the cortex (Fig. 1I).

### Aliphatic and aromatic suberin in the barley endodermis

**Fig. 2.**
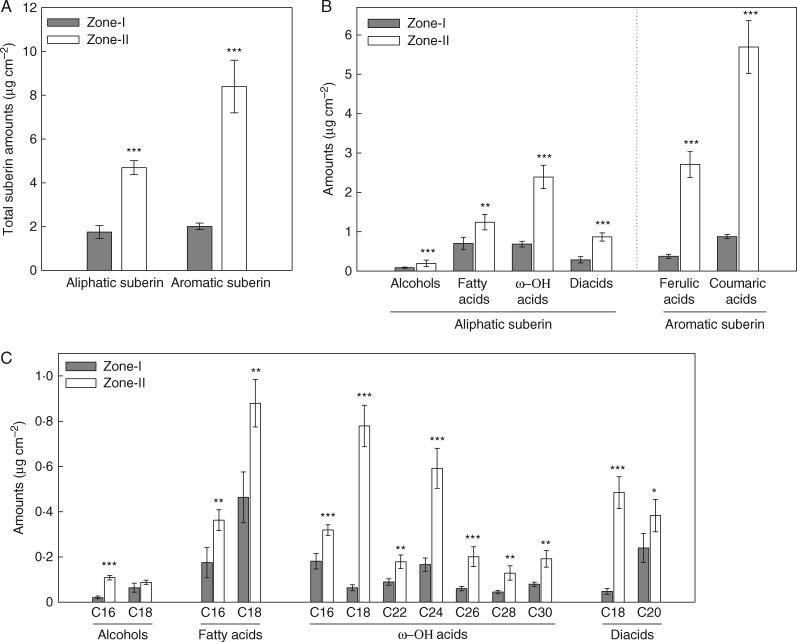
Total amounts (A), substance classes (B) and monomer compositions (C) of aliphatic and aromatic suberin in the stele of 16- to 20-day-old barley seminal roots. Analyses were done for two root zones: Zone-I was the younger zone that included the root apex, and Zone-II was the mature zone, including laterals, towards the base. Enzymatically digested and solvent-extracted root cell walls were subjected to BF_3_/MeOH transesterification and analysed using gas chromatography and mass spectrometry. Absolute amounts are given as means in µg cm^–2^ ± s.d. (*n* = 4 roots). The significant levels of *P* ≤ 0·05, *P* ≤ 0·01 and *P* ≤ 0·001 are indicated by *, ** and ***, respectively (two sample *t*-test).

The whole stele, which included the endodermis, was resistant to enzymatic digestion. The suberin in all of the endodermal samples was composed of two main classes: aliphatic and aromatic suberin (Fig. 2A). The total aliphatic and aromatic suberin contents in the stele of Zone-I (the apical part of the root) were significantly lower than in Zone-II (the basal part of the root) (*P* < 0·001; Fig. 2A). On average, Zone-II had 2·6-fold greater amounts of aliphatic suberin and 4·2-fold greater amounts of aromatic suberin compared with Zone-I. These data were well correlated with the anatomical studies, in which Zone-II had brighter suberin staining than Zone-I (see Fig. 1E, F).

Alcohols, fatty acids, ω-hydroxy acids (ω-OH acids) and diacids were the major substance classes of aliphatic suberin (Fig. 2B). The total amounts of these substances were significantly higher in Zone-II than in Zone-I (*P* < 0·01; Fig. 2B). The largest differences between the zones were observed for ω-hydroxy acids and diacids. Aromatic suberin was mainly composed of ferulic and coumaric acids (Fig. 2B). The ferulic and coumaric acid contents were 6·5-fold greater in Zone-II than in Zone-I.

The chain length distribution of the aliphatic monomers varied from C_16_ to C_30_ (Fig. 2C). Very short chains, such as C_16_ and C_18_, were prominent in all substance classes. The ω-hydroxy acids showed the greatest diversity; carbon chain lengths for these compounds varied from C_16_ to C_30_ (Fig. 2C). Overall, all monomer contents were markedly greater in the mature part of the root (Zone-II) than in the younger part of the root (Zone-I) (*P* < 0·05; Fig. 2C).

### Hydraulic conductivity of the roots

**Fig. 3.**
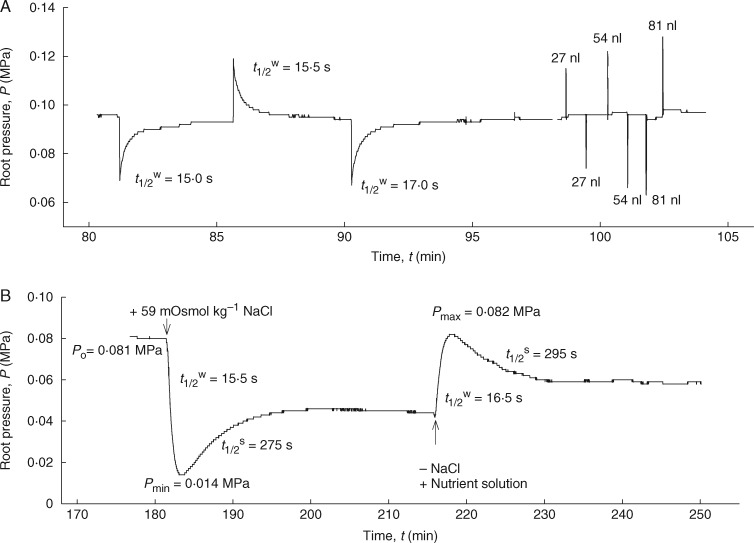
Time course vs. pressure change during a typical experiment with the root pressure probe. A hydroponically grown, 20-day-old barley seminal root (without laterals) was attached to the root pressure probe. (A) Measurements of the hydrostatic pressure relaxations and elastic coefficient (*β*) by moving the metal rod forwards and backwards. (B) Responses of root pressure (*P*_r_) in relation to the change in osmotic pressure in the external medium (either by adding 59 mOsmol kg^–1^ NaCl to the medium or by removing it from the medium). Biphasic responses consist of rapid water efflux (or influx) followed by slow solute influx (or efflux). The addition of NaCl to the medium resulted in a drop of *P*_r_ but it failed to reach the original pressure due to the inhibition of other electrogenic pumps in the plasma membrane.

When connected to the root pressure probe, the seminal roots took 2–3 h to generate steady-state root pressures. Stable pressures varied according to the individual roots, and the mean values ranged between 0·1 and 0·2 MPa. When measured using hydrostatic pressure gradients (Fig. 3A), the hydrostatic *Lp*_r_ of end-segments of seminal roots (Zone-I) was 9·4 × 10^–8^ m s^–1^ MPa^–1^ under well-stirred external root medium conditions (Table 1). The hydrostatic *Lp*_r_ measured with unstirred root medium was 9·7 × 10^–8^ m s^–1^ MPa^–1^. The ratio of well-stirred to unstirred *Lp*_r_ was not significantly different from unity, suggesting that the unstirred layers around the root did not affect the hydrostatic *Lp*_r_. When measured using an osmotic pressure gradient (replacing the nutrient solution with 30 mm NaCl or 59 mOsmol kg^–1^; Fig. 3B), the average osmotic *Lp*_r_ values were 9·5 and 4·2 × 10^–8^ m s^–1^ MPa^–1^ for well-stirred and unstirred conditions, respectively. Although unstirred layers in the external medium had no effect on the hydrostatic *Lp*_r_, they decreased the osmotic *Lp*_r_ by 2·4-fold. When measured under well-stirred conditions, the estimated hydrostatic/osmotic ratios of *Lp*_r_ were approximately at unity, indicating a relatively greater cell to cell water flow in barley roots (Table 1). However, when measured under unstirred conditions, this ratio was increased by 2·6-fold, indicating that the unstirred layers primarily affect osmotic *Lp*_r_ rather than hydrostatic *Lp*_r_.

**Table 1:** Hydraulic conductivity (*Lp*_r_) of end-segments of seminal roots of barley grown in aerated hydroponics for 14–20 d, measured with a root pressure probe

Root medium	Hydraulic conductivity, *Lp*_r_ (10^–8^ m s^–1^ MPa^–1^)		HY:OS ratio
	Hydrostatic (HY)	Osmotic (OS)	
Well-stirred (WS)	9·4 ± 3·1 (*n* = 15)^a^	9·5 ± 3·7 (*n* = 15)^a^	1·1 ± 0·3
Unstirred (US)	9·7 ± 4·2 (n = 6)^a^	4·2 ± 2·6 (*n* = 6)^b^	2·6 ± 0·8
WS/US ratio	1·0 ± 0·1	2·4 ± 0·9	

Hydrostatic and osmotic *Lp*_r_ were measured either rapidly stirring (well-stirred) or without stirring (unstirred) the root medium. Osmotic *Lp*_r_ was measured by replacing the external nutrient solution by 59 mOsmol kg^–1^ NaCl in the nutrient solution.

Values given are means ± s.d. and the numbers of measured roots are in parentheses. Different letters indicate significant differences at the *P* < 0·05 level.

**Fig. 4.**
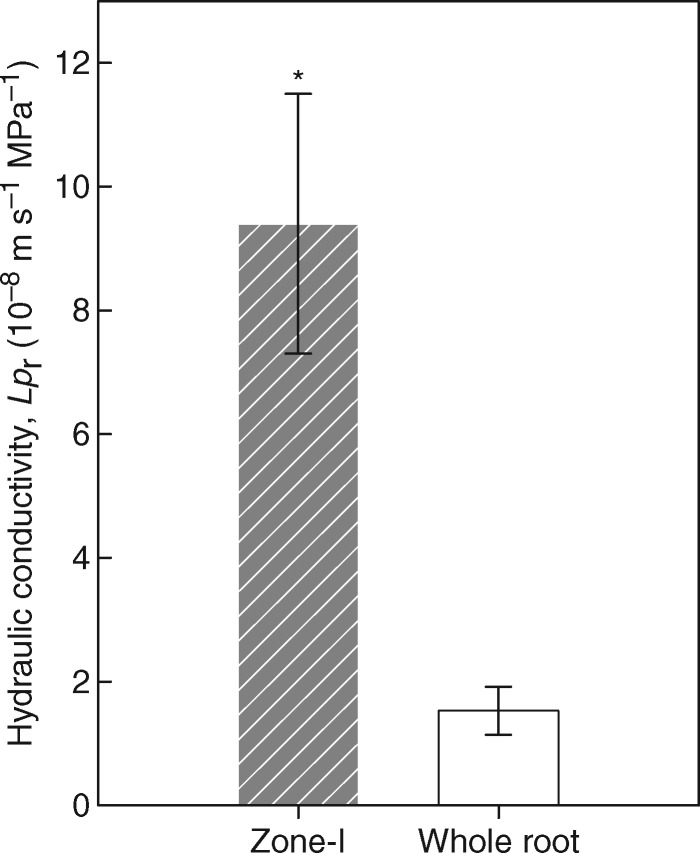
The *Lp*_r_ of the apical zone (Zone-I) and whole roots of 16- to 20-day-old barley seminal roots, grown in aerated hydroponics, and measured with a root pressure probe under well-stirred conditions. The *Lp*_r_ of the whole root is approx. 6-fold smaller than that of the apical zone (Zone-I). Values are the means ± s.d. of ten replicates. A significant difference of *P* ≤ 0·05 is denoted by * (two sample *t*-test).

The comparison of *Lp*_r_ of whole seminal roots and the apical zone (Zone-I) revealed that the younger zone had approx. 6-fold greater water permeability than the whole root (9·4 vs. 1·5 × 10^–8^ m s^–1^ MPa^–1^), which included mature, well-suberized Zone-II (Fig. 4). This indicated that the deposition of SL in the endodermis of the mature/basal zone (Zone-II) significantly decreased the overall radial water permeability of the whole root.

### Water permeability of cell membranes of cortical cells measured by a cell pressure probe

The root cortical cells (unmodified cells in the mid cortex) had turgor pressure values ranging from 0·36 to 0·69 MPa (Table 2). The elastic modulus values of the cells (*ε*) varied from 0·4 to 14 MPa, and the half-times of water exchange (*tc*_1/2_^w^) ranged from 1 to 80 s. The relatively large range of *ε* values is probably due to the variation in cell volume. The actual volume calculations were not possible for the exactly measured cells and instead average cell diameter and length obtained from the root cross-sections of the same place were used for the calculations. Assuming cylindrical cell geometry, the average volume of a cortex cell was 1·3 × 10^–13^ m^3^ (130 pL). There was also a rather large range of *tc*_1/2_^w^ in single cell measurements. At the beginning of the experiment (just after puncturing the cell), seven out of nine cells had a short *tc*_1/2_^w^ ranging from 1 to 6 s, while two cells had *tc*_1/2_^w^ >10 s. Continuous measurements showed that the *tc*_1/2_^w^ increased up to 80 s then recovered to short *tc*_1/2_^w^ within 40–160 min (recovery to the initial values in four cells). A similar phenomenon was observed in corn root cortical cells (Wan *et al.*, 2004). Their results were interpreted as being caused by the tremendous water flux during the puncturing procedure, which acted as a mechanical stress and resulted in the closure of AQPs. Wan *et al.* (2004) demonstrated that about 1 h was needed to recover to short *tc*_1/2_^w^, which are probably the original values. The calculated *Lp*_c_ values were 1·9 × 10^–7^ and 1·5 × 10^–6^ m s^–1^ MPa^–1^ for the minimum and maximum, respectively. When calculating the minimum *Lp*_c_, one cell was excluded, as it did not show any increase in the *tc*_1/2_^w^ during a 15 min long measurement. During calculations of the maximum *Lp*_c_, two cells that showed long *tc*_1/2_^w^ after puncturing were excluded.

**Table 2:** Turgor pressure (*P*), volumetric elastic modulus (*ε*), half times (*t_C_*_1/2_^w^) and hydraulic conductivity (*Lp*_c_) of cortical cells at a distance of 40–50 mm from the root tip

Water relation parameters		Values
Stationary turgor pressure (*P*; MPa)	Range (min – max)	0·36–0·69
	Mean ± s.d.	0·56 ± 0·11 (*n* = 9)
Volumetric elastic modulus (*ε*; MPa)	Range (min – max)	0·4–14
	Mean ± s.d.	3·4 ± 4·8 (*n* = 9)
Half-time of water exchange (*t_C_*_1/2_^w^; s)	Range (min – max)	1–80
Minimum, which is < 10 s	Mean ± s.d.	3·6 ± 1·7 (*n* = 7)
Maximum	Mean ± s.d.	32 ± 22 (*n* = 8)
Hydraulic conductivity (*Lp*_c_; 10^–6^ m s^–1^MPa^–1^)	Range (min – max)	0·013–5·7
Minimum	Mean ± s.d.	0·19 ± 0·16 (*n* = 8)
Maximum	Mean ± s.d.	1·5 ± 2·0 (*n* = 7)

Values given are ranges (minimum and maximum), and means ± s.d.

Volumes of cells were calculated assuming that they were cylindrical.

A mean value of 1·3 × 10^–13^ m^3^ (130 pL) was taken to calculate *ε* and *Lp*_c_.

Numbers of measured cells are in parentheses.

### Root hydraulic conductivity as calculated from the hydraulic conductivity of cortical cells

According to the earlier work of Steudle and Jeschke (1983) and Steudle and Brinckmann (1989), average values of cell *Lp*_c_ were used to estimate the root *Lp*_r_ values of corn. This estimation assumed that the radial flow of water across the root cylinder was due solely to cell to cell movement through the plasma membrane, excluding apoplastic passage. The root was assumed to consist of parallel concentric rings of membranes. In this model, the overall root *Lp*_r_ relates to the *Lp*_c_ values of individual cells by the following equation:

(7) $$\frac{1}{Lp_{r}}=\frac{1}{Lp}\sum_{i=1}^{n}\frac{r_{o}}{r_{i}}.$$

In this equation, *r*_o_ refers to the outer radius of roots (300 μm), and *r*_i_ refers to the radii of the *n* membrane cylinders to be crossed (six layers including the endodermis, which equates to *n* = 12 concentric rings of plasma membranes). The equation assumes that the hydraulic resistances of individual cell layers arranged in series are additive and that cell membranes must be crossed twice per layer. For geometric reasons, the inner rings contribute more to the overall resistance than the outer rings. Both the rhizodermis and cortex, including the endodermis, were considered in these calculations. For the endodermal resistance, it is believed that only the passage cells (around 80 % of the total endodermal cells in Zone-I) contribute to the water flow, which is a realistic assumption. However, in this calculation for barley, it was assumed that all the endodermal cells contributed to the water flow through membranes. For the roots used here, the average Σ*r*_o_/*r*_i_ was 21, and the average *Lp*_c_ (max) = 1·5 × 10^–6^ m s^–1^ MPa^–1^, which resulted in *Lp*_r_ = 7·1 × 10^–8^ m s^–1^ MPa^–1^. However, the calculated *Lp*_r_ value should be considered the upper limit due to the exclusion of the endodermal resistance.

### Root axial resistance for water transport

**Fig. 5.**
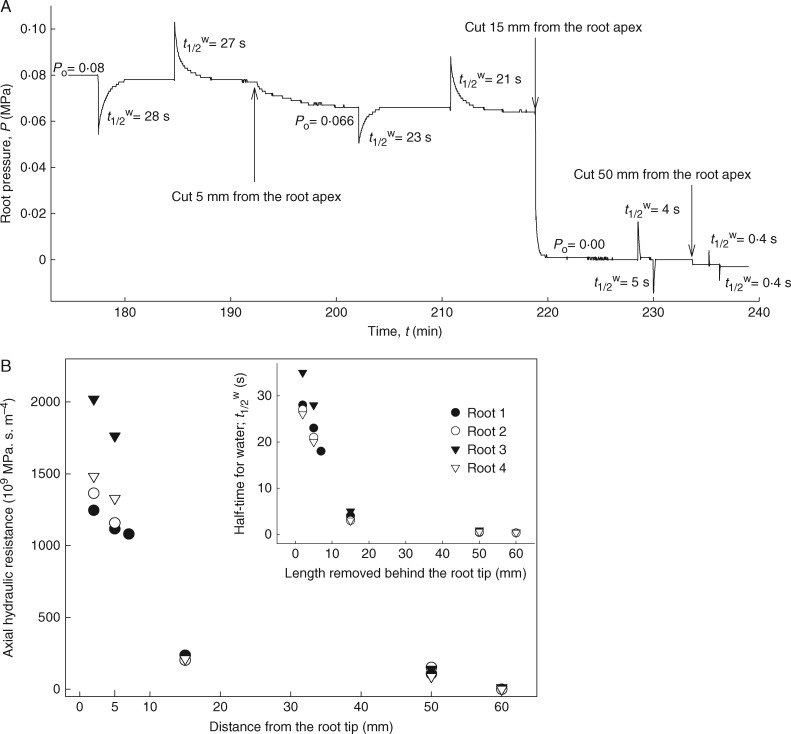
Cutting experiment on an excised maize root attached to the root pressure probe. When the root was successively cut with a razor blade, starting at the apex, the root pressure dropped immediately when developed xylem was cut. Between cuts, hydrostatic pressure relaxations were performed in order to measure changes in the hydraulic resistance of the root. The cutting experiments also allowed the longitudinal hydraulic resistance in the root to be estimated and provided information about the tightness of the silicone seal. In the pressure probe measurement, it is assumed that the radial resistance is significantly greater than the axial resistance to overall water flow in roots. The cutting experiment demonstrated that this is true. When the root apical end of a 5 mm long segment (including the root tip) was removed, the root pressure (*P*_r_) and the *t*_1/2_^w^ dropped by 20 % (A), indicating that some early metaxylem vessels are partially matured and semi-conductive at this distance from the tip. However, as soon as the fully matured and functional early metaxylem vessels (starts at approx. 15 mm from the apex) were cut, the *P*_r_ immediately dropped to zero and the *t*_1/2_^w^ decreased by 6- to 7-fold due to leaking out of the open xylem vessels. A further cut at 50 mm from the apex, where the central late metaxylem vessel (central duct) is conductive, resulted in decreasing the *t*_1/2_^w^ to zero. There is a direct and positive correlation between the *t*_1/2_^w^ and the axial hydraulic resistance of the root (xylem vessels) (B). The experimental data proved that the measured *t*_1/2_^w^ and *Lp*_r_ refer to the radial transport of water into the roots (from the external medium to the root xylem) but not to the axial transport along the root xylem vessels.

In the pressure probe measurement, it is assumed that the radial resistance is significantly greater than the axial resistance to overall water flow in roots. The cutting experiment demonstrated that this is true. When the root apical end of a 5 mm long segment (including the root tip) was removed, the root pressure (*P*_r_) and the *t*_1/2_^w^ dropped by 20 % (Fig. 5A), indicating that early metaxylem vessels (the number can vary between seven and nine), which locate around the central duct/late metaxylem, are partially matured and semi-conductive at this distance from the tip. It is likely that these vessels still contain debris of cell content which made a detectable resistance to the axial water flow. However, as soon as the mature and fully functional early metaxylem vessels (starts at approx. 15 mm from the apex) were cut, the *P*_r_ immediately dropped to zero and the *t*_1/2_^w^ decreased by 6- to 7-fold due to leaking out of the open xylem vessels. Nevertheless, even at this stage, the *t*_1/2_^w^ was not zero. A further cut at 50 mm from the apex, where the fully developed central late metaxylem appeared (Fig. 1D, E), resulted in decreasing the *t*_1/2_^w^ close to zero due to complete leak out of xylem vessels (negligible axial resistance). There is a direct and positive correlation between the *t*_1/2_^w^ and the axial hydraulic resistance of the root (xylem vessels) (Fig. 5B). The experimental data proved that the measured *t*_1/2_^w^ and *Lp*_r_ refer to the radial transport of water into the roots (from the external medium to the root xylem) but not to the axial transport along the root xylem vessels.

### Solute permeability and reflection coefficient of roots

**Fig. 6.**
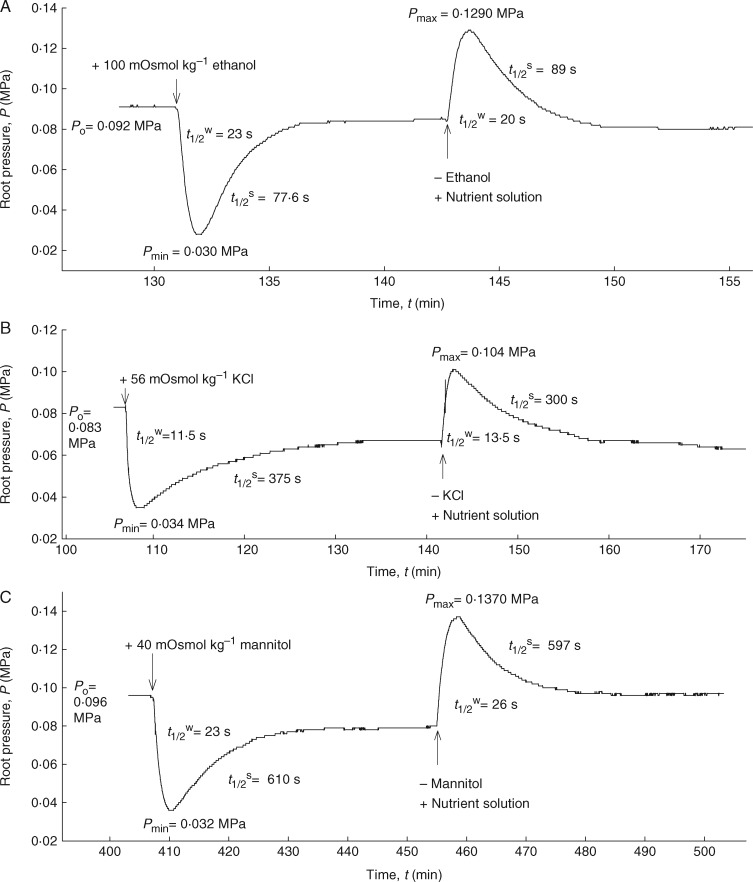

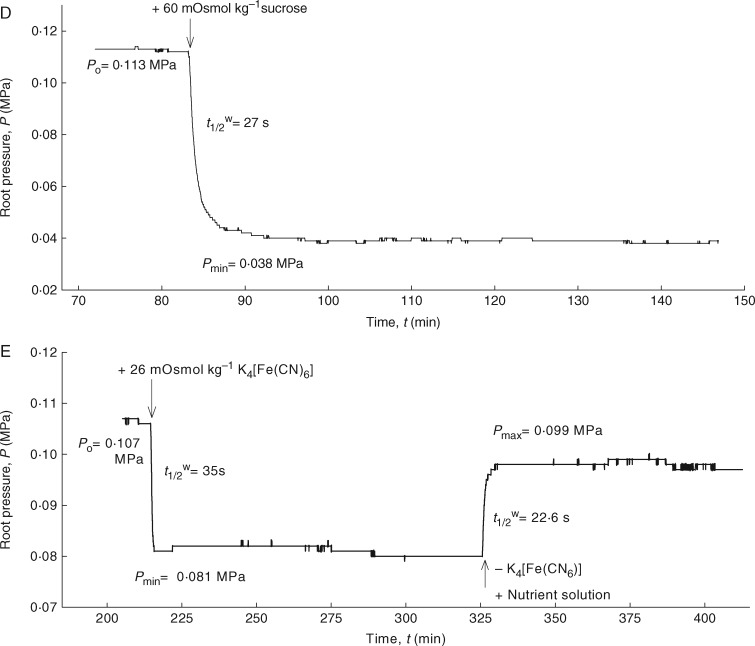
Measurement of solute permeabilities (*P*_sr_) and reflection coefficients (*σ*_sr_) of seminal roots of barley for different test solutes using the root pressure probe in well-stirred conditions. Responses of root pressure (*P*_r_) in relation to the change in osmotic pressure in the medium either by adding 100 mOsmol kg^–1^ ethanol (A), 56 mOsmol kg^–1^ KCl (B), 40 mOsmol kg^–1^ mannitol (C), 60 mOsmol kg^–1^ sucrose (D) and 26 mOsmol kg^–1^ K_4_[Fe(CN)_6_] (E) or by removing them from the medium. The addition of ethanol, KCl and mannitol to the medium resulted in biphasic responses due to rapid efflux of water, followed by slow influx of solutes. In contrast, addition of sucrose and K_4_[Fe(CN)_6_] to the external medium gave monophasic responses in which the second phase or solute influx (solute permeation into the root) is missing.

Once the roots had achieved steady-state pressures, the osmotic pressure of the medium was changed by adding different osmotic solutes, which were rapidly permeating (ethanol, NaCl and KCl), less rapidly perneating (mannitol) or virtually non-permeating (sucrose and K_4_[Fe(CN)_6_]) the membranes. Hypertonic conditions caused an efflux of water, whereas hypotonic conditions caused an influx. However, for the permeating solutes, there was a reverse influx (efflux) of solutes into (and out of) the root, which was denoted as a ‘solute phase’ (second phase of the biphasic relaxations; Figs 3B and 6A–C). Following a minimum (or a maximum) in pressure, the root pressure was expected to return to the baseline value. With the roots used here, however, the original root pressure was nearly but not fully recovered in the solute phase with ethanol, NaCl and KCl. This observation suggests a possible inhibition of the plasma membrane ion pumps at high solute concentrations, as has previously been found in corn (Steudle *et al.*, 1987; see the Discussion). For non-permeating solutes, such as sucrose and K_4_[Fe(CN)_6_], the pressure relaxations were monophasic (Fig. 6D, E). The solute phase was either lacking or very long due to the long half-times caused by low *P*_sr_ [eqn (4)]. In contrast to previous results in corn (Steudle *et al.*, 1987), osmotic experiments with mannitol showed biphasic relaxations in the young barley roots, indicating a measurable uptake of this solute (Fig. 6C). Determination of the *P*_sr_ and *σ*_sr_ values for the different solutes using eqn (4) or eqn (5) required the estimation of the volume of the functioning xylem, which was obtained from the root cross-sections.

The results indicated a marked difference between the *P*_sr_ values of barley roots for different solutes (Table 3). Most interesting was the fact that K_4_[Fe(CN)_6_] did not permeate at all, similar to previous observations by Ranathunge *et al.* (2005) in rice. The *P*_sr_ values for NaCl and KCl were 4·5-fold lower than that of ethanol, which is lipophilic and rapidly permeated through the plasmalemma (Steudle and Peterson, 1998; Miyamoto *et al.*, 2001; Meyer *et al.*, 2011). However, values of *P_sr_* for NaCl and KCl were significantly higher than that of mannitol (*F*_3,40_ = 159·89; *P* < 0·0001). The *P*_sr_ values for sucrose (which has a larger molecular weight than mannitol) and K_4_[Fe(CN)_6_] were not measurable because the solute phase was missing for these compounds (second phase in the osmotic experiments; Fig. 6D, E).

**Table 3:** Solute permeability (*P*_sr_) and reflection coefficient (*σ*_sr_) of end-segments of seminal roots of barley, grown in aerated hydroponics for 14–20 d, measured with a root pressure probe

Type of solute	Solute permeability (*P*_sr_; 10^–9^ m s^–1^)	Reflection coefficient (*σ*_sr_)	
		Measured	Corrected
Ethanol	12·5 ± 2·4^a^	0·23 ± 0·11^a^	0·35 ± 0·19^a^
NaCl	2·8 ± 0·5^b^	0·51 ± 0·09^b^	0·69 ± 0·12^b^
KCl	2·5 ± 0·3^b^	0·52 ± 0·06^b^	0·68 ± 0·10^b^
Mannitol	1·7 ± 0·4^c^	0·71 ± 0·09^c^	0·90 ± 0·16^c^
Sucrose	n.m.	0·45 ± 0·10^b^	–
K_4_[Fe(CN)_6_]	n.m.	0·61 ± 0·20^bc^	–

Values given are means ± s.d. for tenroots.

Different letters in the same column indicate significant differences at the *P* < 0·05 level.

n.m., not measurable.

The measured values of *σ*_sr_ for barley roots ranged from 0·2 to 0·7, depending on the solute used (Table 3). The lowest *σ*_sr_ was observed for ethanol, which was the solute that permeated most rapidly through the root cylinder. This value was 2-fold smaller than the *σ*_sr_ values of NaCl and KCl, and >3-fold smaller than that of mannitol (*F*_5,41_ = 20·24; *P* < 0·0001). However, non-permeating solutes, such as sucrose and K_4_[Fe(CN)_6_], had unexpectedly lower *σ*_sr_ values, which are normally at unity for the plasmalemma (an ideally semi-permeable membrane). The *σ*_sr_ value for sucrose was in the same range as those of NaCl and KCl, while the *σ*_sr_ value for K_4_[Fe(CN)_6_] was located between those of mannitol and sucrose (Table 3). The *σ*_sr_ values, when corrected for solute flow, were approx. 20–35 % greater than the ‘raw’ data of uncorrected ratios of Δ*P*_r_/Δ*π*°_s_ (Table 3). These corrected values showed that mannitol had the greatest *σ*_sr_ value, while ethanol had the lowest. The *σ*_sr_ values for NaCl and KCl were greater than that of ethanol but smaller than that of mannitol {the *σ*_sr_ values for sucrose and K_4_[Fe(CN)_6_] could not be corrected). Although the *σ*_sr_ values increased as *P*_sr_ decreased, they were below unity for those solutes that had virtually zero permeability for semi-permeable membranes.

## DISCUSSION

For the first time, we have investigated the role of suberized barriers, a part of the apoplast, for water and solute transport of barley seminal roots. The findings showed that these barriers negatively affect the overall radial water and solute transport of barley roots. The basal zone with a greater content of suberin exhibited markedly lower permeability for water than the apical zone with a lower content of suberin. The contribution of the root apical zone for overall water transport is significantly greater than the contribution of the basal zone. In the former, the relative contribution of the cell to cell path is greater for water transport than its associated apoplast, which is similar to the case for *A. thaliana* (Ranathunge and Schreiber, 2011). However, the water transport through the apoplast is not negligible.

In roots, suberized cell walls in the endo- and exodermis form transport barriers to water and solutes (Peterson and Cholewa, 1998). In barley, when grown in hydroponics, roots develop no exodermis, even in their basal zones, which is in agreement with the previous finding of Robards *et al.* (1973). This anatomical feature is different from other monocot plants such as rice, onion and *Iris germanica* (Zimmermann and Steudle, 1998; Ranathunge *et al.*, 2005; Meyer *et al.*, 2011). Hydroponically grown corn plants developed a patchy exodermis which resulted in a greater suberin content of the root periphery (Barrowclough *et al.*, 2000; Zimmermann *et al.*, 2000; Schreiber *et al.*, 2005). Barley roots developed an endodermis, where early deposition of CBs appeared as ‘dots’ 20 mm from the root apex (Fig. 1B), which is similar to soybean (Ranathunge *et al.*, 2008) and other angiosperm plants (Ma and Peterson, 2003, and references therein). With maturity, these bands extended in the anticlinal walls (Fig. 1D) which probably added a greater resistance to the apoplastic permeability of the endodermis. SL deposited further back from the apex (60 mm) and initially exhibited as a patchy structure. At the base of Zone-I, SL were not deposited in the complete ring of the endodermis (Fig. 1E). Only further back at the base of the root (100 mm) did SL develop as a complete ring which had approx. 10 % of passage cells without lamellae, (Fig. 1F). The passage cells without SL facilitated the water and solute transport through the transcellular and symplastic components of the cell to cell pathway, but still present a barrier for apoplastic transport due to the presence of CBs.

The absence of an exodermis suggests that the endodermal suberin mostly accounted for the total suberin of barley roots, whereas, faintly stained, diffuse suberin in the rhizodermis accounted for the rest. The total root suberin is made up from aliphatic and aromatic components (Fig. 2A, B), similar to other monocot plants such as rice (Kotula *et al.*, 2009; Ranathunge *et al.*, 2011) and corn (Zimmermann *et al.*, 2000). Even though, the basal zone (Zone-II) had markedly greater amounts of both aliphatic and aromatic suberin than the apical zone (Zone-I), the difference was more pronounced for the aromatic suberin (Fig. 2A). This might be due to some associated aromatics/polyphenolics in the strongly lignified stele of the basal zone of barley roots. The most abundant aliphatic monomers were ω-hydroxy fatty acids, α,ω-dicarboxylic acids and primary carboxylic acids, whereas aromatics were composed of ferulic and coumaric acids, of which the latter is prominent (Fig. 2B). These monomers are also known to be typical components of suberin lamellae in other plant species (Matzke and Riederer 1991; Schreiber *et al.*, 1999; Kolattukudy, 2001; De Simone *et al.*, 2003; Franke *et al.*, 2005; Kotula *et al.*, 2009). However, barley endodermis contained markedly greater amounts of aliphatic suberin than corn endodermis, depending on the root zone (for comparison, see Fig. 3A and Schreiber *et al.*, 2005). On average, differences were approx. 9-fold for Zone-I (apical part) and 4-fold for Zone-II (basal part). For the first time, this study also shows the whole spectrum of monomer chain length distribution in the endodermis of barley roots. Short chain lengths, such as C_16_ and C_18_, dominate in the endodermis (Fig. 2C). More lipophilic very long chains (C_26_, C_28_ and C_30_) were only found in the ω-hydroxy fatty acids (Fig. 2C). In comparison with rice and corn, the aliphatic suberin in barley has a less diverse monomer composition. Such interspecies differences are likely to play a major role in the differences of water and solute transport of roots.

For barley roots, the ratio of measured *Lp*_r_ using a hydrostatic pressure gradient (water flow through the apoplast as well as cell to cell path) and an osmotic gradient (water flow through the cell to cell path) was a little over unity. This suggests a dominant cell to cell water flow rather than a porous apoplastic bypass flow (Steudle and Peterson, 1998). This result can be interpreted using the composite transport model (Steudle and Peterson, 1998), i.e. in the absence of a hydrostatic pressure gradient, the apoplastic path is inefficient due to its low reflection coefficient. As such, water will flow predominantly via the protoplastic (cell to cell) path in response to an osmotic driving force. Further, comparisons between the cell *Lp*_c_ and root *Lp*_r_ of Zone-I also supported the view that the cell to cell pathway provides a greater contribution to the overall radial water flow than the apoplastic pathway. Although this finding was in agreement with the earlier results of Steudle and Jeschke (1983), concerns about the importance of each pathway for overall water flow still remained. On average, 20 % of endodermal cells contained the SL in the root segments (Zone-I) used for the measurements. The deposition of lamellae between the primary cell walls and the plasmalemma is known to reduce the water flow through the plasma membrane (Steudle and Peterson, 1998). Therefore, only those remaining cells without lamellae (passage cells) allowed water to move freely through the endodermis. Assuming that no water moved through the apoplast, the theoretically calculated overall root *Lp*_r_ of Zone-I from the cell *Lp*_c_ (not including the SL resistance) was 26 % lower than the measured root *Lp*_r_ value. This result suggests that there are some apoplastic bypasses in the cortex, which may represent the difference between the measured and calculated values of *Lp*_r_. However, the theoretically calculated *Lp*_r_ should be the upper limit and it should even be smaller if the resistance of suberized endodermal cells (20 % of the total endodermis) accounted for the calculations. This hypothesis of Steudle and Jeschke (1983) was further supported by a recent study by Knipfer *et al.* (2011), in which the closure of AQPs by HgCl_2_ resulted in a reduction of root *Lp*_r_ values by 53 %. Nevertheless, there is still a substantial cell to cell component similar to the results of Steudle and Jeschke (1983). The results of this study conclude that the cell to cell water transport is more pronounced in barley roots, as found for *A. thaliana* (Ranathunge and Schreiber, 2011).

The rate of water transport into the shoot is usually determined by (1) the radial water transport across roots from the soil solution to the root xylem crossing different complex cell layers, including the endodermis, and (2) axial water transport inside the root xylem. It is known that the major hydraulic resistance or rate-limiting step for water transport into the shoot is the former but not the axial/longitudinal resistance in the xylem (Steudle and Peterson, 2000). Usually, the mature and dead xylem vessels/ducts provide exceedingly low resistance to the axial water flow (Steudle and Peterson, 2000). Using the pressure probe technique, we have demonstrated that this is true for the conductive part of Zone-I of barley roots in which the early metaxylem vessels and central late metaxylem vessel are fully matured (Fig. 5). In these roots, even though the column of central metaxylem cells can be identified much earlier, closer to the root apex, these cells fully mature much later, and the distance from the apex depends on the growth conditions (Heimsch, 1951; Lux, 1981). The undifferentiated and immature xylem vessels in the very apical region of Zone-I (at least up to 15 mm) provide some resistance to the axial water flow in the xylem. Hence, the axial hydraulic resistance in the very apical part of Zone-I cannot be neglected. However, in our *Lp*_r_ measurements, this very apical part or ‘hydraulically isolated zone’ was not accounted for in the calculation. This experiment demonstrates that the measured *Lp*_r_ data refer to the radial transport of water in barley roots but not to the axial transport in the xylem vessels.

In addition to water uptake, roots serve as the primary site for the uptake of nutrients and other solutes by plants. In barley roots, there are significant differences between the permeability (*P*_sr_) of electrolytes, such as nutrient salts and non-electrolytes, depending on their size and molecular structure. Small, lipophilic molecules (i.e. ethanol) move faster crossing the plasma membranes of the root than larger molecules with or without charges. These flows may also differ among different plant species, according to the composite transport model of roots (Steudle *et al.*, 1987; Frensch and Steudle, 1989; Steudle and Peterson, 1998; Martinka *et al.*, 2014). Differences between species may be caused by differences in root morphological and anatomical structures. For example, corn roots contained 12–14 cortical cell layers in series in the cylinder (Ye and Steudle, 2006), while barley contained 4–5 layers. The stele of barley was completely lignified with thick walls and densely packed cells containing no apparent air spaces. Only those cells surrounding the xylem were lignified in corn. As expected, barley roots had very low or no permeability for K_4_[Fe(CN)_6_] (Fig. 6E). It has been previously demonstrated that this salt with four negative charges moves slowly in the apoplast of corn and rice roots. The ferrocyanide anion is known to be repelled by the fixed negative charges of the cell walls (Ranathunge *et al.*, 2005). There were also differences between barley and corn for sucrose, the non-electrolyte with the largest molar weight in the experiment. Sucrose permeated slowly but significantly across the root cylinder of corn during a very long treatment time of 4–5 h (Steudle *et al.*, 1987). In barley, however, sucrose did not move into the stele for a period of 1·5–2 h (Fig. 6D). Longer experiments with sucrose (>2 h) resulted in a gradual decline of the root pressure, most probably due to some degree of membrane leakage. Conversely, mannitol slowly permeated into the roots of barley (Fig. 6C) but did not move across the roots of corn (Steudle *et al.*, 1987). However, in corn, the authors conducted the experiment only for a short period of time (approx. 40 min) and they probably did not wait long enough to observe the permeation of mannitol into the root. The permeability of the roots for ethanol was similar for both species (Fig. 6A). This result would be expected because this lipophilic solute rapidly crosses the plasmalemma (Steudle and Henzler, 1995; Henzler *et al.*, 2004). In summary, if barley roots behaved as perfect osmometers and were impermeable for all the solutes, biphasic responses would not have been seen for the permeable solutes, such as ethanol, KCl, NaCl and mannitol. In contrast, larger molecules with or without charges, such as sucrose and K_4_[Fe(CN)_6_], failed to permeate across the root. These results indicate that *P*_sr_ of barley roots depends on the solute used, and the roots are not perfect osmometers.

Reflection coefficients (*σ*_sr_) smaller than unity have been found for many roots using different techniques (Steudle and Peterson, 1998). It has been stressed many times in the literature that an *σ*_sr_ value is not a direct measure of the *P*_sr_ of a root, although these values sometimes correlate such that a low *σ*_sr_ may correspond to a high *P*_sr_ (Steudle and Peterson, 1998). The correlation may be simple and straightforward for uniform, homogeneous membranes (see figs 3–12 in Nobel, 1999) but may be complicated in composite membrane systems (Kedem and Katchalsky, 1963*a*, *b*). According to Kedem and Katchalsky (1958), the definition of semi-permeability holds if *σ*_sr_ = 1 and *P*_sr_ = 0 at the same time. Further, by definition, *P*_sr_ = 0 and *Lp*_r_ > 0 should also hold for a semi-permeable barrier. However, in barley, the measured values of root *P*_sr_ > 0 and *σ*_sr_ < 1 revealed that they do not behave like an ideal osmometer for the tested solutes.

Similarly to root *Lp*_r_ and *P*_sr_ values, the *σ*_sr_ value may be subjected to errors due to unstirred layers (USLs) both inside and outside of the root (Ye and Steudle, 2006; Knipfer *et al.*, 2007; Knipfer and Steudle, 2008). Under well-stirred conditions, the estimated effect of internal USLs on the *σ*_sr_ values of young corn roots was as small as 7 % (Steudle and Frensch, 1989). For the thinner roots of barley, this effect should be even smaller. The impact of USLs was minimized by vigorously stirring the medium surrounding the root, but stirring cannot affect the USLs within the root. When measured in the stagnant solution, the effects of USLs were substantial in the osmotic experiments (Table 1). External USLs around the root reduced osmotic *Lp*_r_ values by 2·4-fold compared with the well-stirred conditions. In contrast, USLs failed to reduce hydrostatic *Lp*_r_ values below those of well-stirred conditions. This result is understandable because water flow driven by a hydrostatic pressure gradient should not be affected, as has been previously described by Steudle and Frensch (1989). In agreement with the present study, Knipfer and Fricke (2010) also found that root *σ*_sr_ values for solutes were significantly smaller when measured in a stagnant medium.

In young barley roots, the cortical apoplast allows solutes to diffuse up to the endodermis. Therefore, the cortical apoplast forms an internal USL. The cortex should have a rather low *σ*_sr_, which may be even close to zero in the presence of a rather high *P*_sr_. For a composite structure consisting of an endodermis (the innermost modified cortical cell layer with rather low *P*_sr_ and high *σ*_sr_) and other cortical cells arranged in series, the basic principles of irreversible thermodynamics propose that the cortex should contribute to the overall *σ*_sr_ according to its permeability for the solute, which should be high (Kedem and Katchalsky, 1963*a*, *b*). Denoting the cortex with the superscript ‘cor’ and the endodermis with ‘en’, the overall *σ*_sr_ is described by the following equation:

(8) $$\sigma_{sr}=\sigma_{sr}^{cor}\frac{P_{sr}}{P_{sr}^{cor}}+\sigma_{sr}^{en}\frac{P_{sr}}{P_{sr}^{en}}\;,\;where\;\;\frac{1}{P_{sr}}=\frac{1}{P_{sr}^{cor}}+\frac{1}{P_{sr}^{en}}.$$

Therefore, roots may exhibit a low *σ*_sr_ in the presence of a low *P*_sr_. For tree roots, *σ*_sr_ values were as low as 0·2–0·4. At the same time, the root *P*_sr_ value was too small to be measured by the pressure probe (Steudle and Peterson, 1998). Similarly, in barley, the *σ*_sr_ values were substantially smaller than unity for K_4_[Fe(CN)_6_] and sucrose (which were virtually non-permeating). Alternatively, as suggested by Knipfer and Fricke (2010), if roots have a value of *P*_sr_^en^ = 0 (and a value of *P*_sr_ = 0), the contribution of a USL (due either to the cortex or to an adjacent layer in the medium) should be small. Under these conditions, it holds that *P*_sr_/*P*_sr_^cor^ ≅ 0 and *P*_sr_/*P*_sr_^en^ ≅ 1. However, in barley, it was not the case and *P*_sr_^en^ was not negligible. If both the apoplastic and the cell to cell permeability of ions in the endodermis are considered, the apoplastic component may be small but not negligible. Kronzucker and Britto (2011) demonstrated the importance of apoplastic bypasses of solutes in the endodermis compared with the plasma membrane permeability for sodium.

In conclusion, the data show that suberized cell walls make strong barriers for water transport in barley roots, especially in the basal zones. Barley roots did not form an exodermis, and the endodermal suberin accounted for the total root suberin. The absolute suberin amount in the basal zone was significantly higher than in the apical zone, which was inversely proportional to the *Lp*_r_. Comparison of the *Lp*_c_ and *Lp*_r_ of the apical zone indicated that the results were comparable and suggested a dominant cell to cell transport of water. It is likely that the passage cells without SL facilitate a significantly greater water flow through the endodermis, at least in the younger zone. At the root level, there was also some apoplastic bypass of water in the cortex. The xylem resistance for axial water flow depends on the xylem maturation. When the xylem is fully matured and functional, the axial hydraulic resistance dropped to virtually zero (Fig. 5). The permeability of barley roots for solutes depends on their size, molecular structure and root anatomy, and relates to the proposed composite transport model. The data support the hypothesis that water and solute transport across barley roots is composite in nature. The composite transport model should be extended to include serial arrays of components (cortex, endodermis) alongside the parallel components (apoplast, cell to cell).

## SUPPLEMENTARY DATA

Supplementary data are available online at www.aob.oxfordjournals.org and consist of the following. Figure S1: schematic diagram showing the development of Casparian bands (CBs) and suberin lamellae (SL) of apical (Zone-I) and basal (Zone-II) zones of barley seminal roots, grown in aerated hydroponics for 16–20 d.

## Supplementary Material

Supplementary Data
