# Supplementary material for: The composite water and solute transport of barley (Hordeum vulgare) roots: effect of suberized barriers
Source: Ann Bot. 2017 Jan 8;119(4):629–43. doi: 10.1093/aob/mcw252 (PMC5604597; doi:10.1093/aob/mcw252)
Supplement: Supplementary Data [file mcw252_Supp.ppt]

## Slide 1
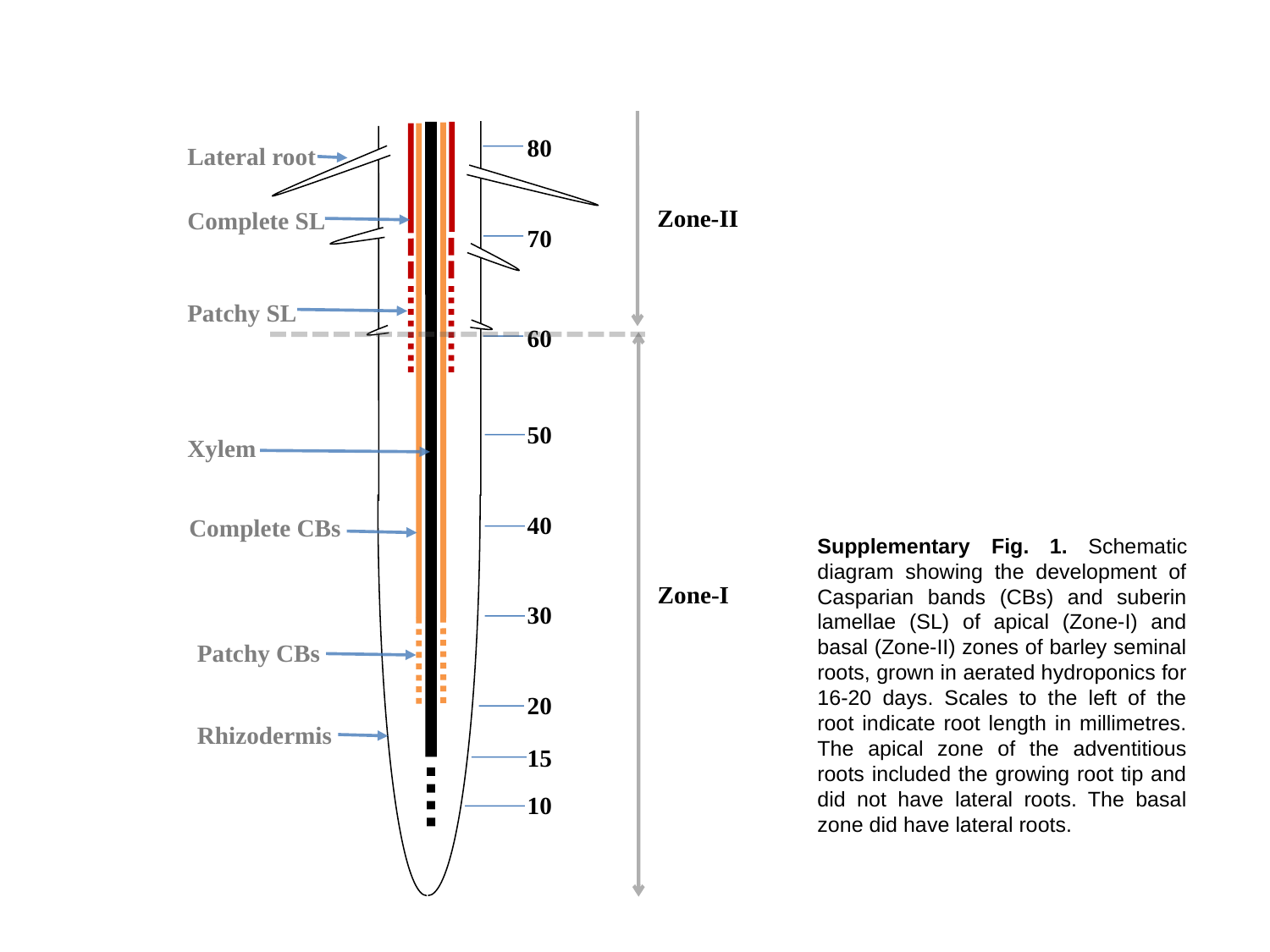

80
70
60
50
40
30
20
15
10
Lateral root
Zone-II
Complete SL
Patchy SL
Xylem
Complete CBs
Zone-I
Patchy CBs
Rhizodermis
Supplementary Fig. 1. Schematic diagram showing the development of Casparian bands (CBs) and suberin lamellae (SL) of apical (Zone-I) and basal (Zone-II) zones of barley seminal roots, grown in aerated hydroponics for 16-20 days. Scales to the left of the root indicate root length in millimetres. The apical zone of the adventitious roots included the growing root tip and did not have lateral roots. The basal zone did have lateral roots.
